# Validation of a Blood-Based Protein Biomarker Panel for a Risk Assessment of Lethal Lung Cancer in the Physicians’ Health Study

**DOI:** 10.3390/cancers16112070

**Published:** 2024-05-30

**Authors:** Lulu Song, Ehsan Irajizad, Andrew Rundle, Howard D. Sesso, John Michael Gaziano, Jody V. Vykoukal, Kim-Anh Do, Jennifer B. Dennison, Edwin J. Ostrin, Johannes F. Fahrmann, Frederica Perera, Samir Hanash

**Affiliations:** 1 Department of Biostatistics, The University of Texas MD Anderson Cancer Center, Houston, TX 77030, USA; lsong3@mdanderson.org (L.S.); eirajizad@mdanderson.org (E.I.); kimdo@mdanderson.org (K.-A.D.); 2 Department of Epidemiology, Mailman School of Public Health, Columbia University, New York, NY 10032, USA; agr3@cumc.columbia.edu; 3 Divisions of Preventive Medicine and Aging, Department of Medicine, Brigham and Women’s Hospital, Boston, MA 02215, USA; hsesso@bwh.harvard.edu (H.D.S.); jmgaziano@bwh.harvard.edu (J.M.G.); 4 Department of Epidemiology, Harvard T.H. Chan School of Public Health, Boston, MA 02115, USA; fpp1@cumc.columbia.edu; 5 Massachusetts Veterans Epidemiology Research and Information Center (MAVERIC), VA Boston Healthcare System, Boston, MA 02115, USA; 6 Department of Clinical Cancer Prevention, The University of Texas MD Anderson Cancer Center, Houston, TX 77030, USA; jvykouka@mdanderson.org (J.V.V.); jffahrmann@mdanderson.org (J.F.F.); 7 Department of Pulmonary Medicine, The University of Texas MD Anderson Cancer Center, Houston, TX 77030, USA

**Keywords:** lung cancer, biomarkers, physicians’ health study

## Abstract

**Simple Summary:**

Improvements in lung cancer risk assessment to inform on the need for screening may be achieved through the use of biomarkers. Here, we report the findings of a validation study of a panel of four circulating protein biomarkers for the risk prediction of lung cancer in a cohort of pre-diagnostic plasmas obtained from the Physician’s Health Study (PHS). We demonstrate that the protein panel can identify individuals at high risk of lung cancer up to two years prior to clinical diagnosis.

**Abstract:**

This study aimed to assess a four-marker protein panel (4MP)’s performance, including the precursor form of surfactant protein B, cancer antigen 125, carcinoembryonic antigen, and cytokeratin-19, for predicting lung cancer in a cohort enriched with never- and ever-smokers. Blinded pre-diagnostic plasma samples collected within 2 years prior to a lung cancer diagnosis from 25 cases and 100 sex-, age-, and smoking-matched controls were obtained from the Physicians’ Health Study (PHS). The 4MP yielded AUC performance estimates of 0.76 (95% CI: 0.61–0.92) and 0.69 (95% CI: 0.56–0.82) for predicting lung cancer within one year and within two years of diagnosis, respectively. When stratifying into ever-smokers and never-smokers, the 4MP had respective AUCs of 0.77 (95% CI: 0.63–0.92) and 0.72 (95% CI: 0.17–1.00) for a 1-year risk of lung cancer. The AUCs of the 4MP for predicting metastatic lung cancer within one year and two years of the blood draw were 0.95 (95% CI: 0.87–1.00) and 0.78 (95% CI: 0.62–0.94), respectively. Our findings indicate that a blood-based biomarker panel may be useful in identifying ever- and never-smokers at high risk of a diagnosis of lung cancer within one-to-two years.

## 1. Introduction

Low-dose computed tomography (LDCT) has been shown to be effective in reducing mortality due to lung cancer (LC) [1,2,3]. The National Lung Screening Trial (NLST) was the first randomized controlled study to show the mortality benefit from lung cancer screening by LDCT: it yielded a reduction in lung cancer death by ~20% compared to chest radiography amongst individuals with a significant history of cigarette smoking [1,2]. Other trials such as the European NELSON trial [3], the Multicentric Italian Lung Detection (MILD) [4], and the German Lung cancer Screening Intervention (LUSI) [5] helped to validate the NLST findings.

Currently, the United States Preventive Task Force (USPSTF) recommends LDCT screening for individuals aged equal to or greater than 50 with a 20 or more pack-year history of cigarette smoking and who are actively smoking or quit within the last 15 years [4]. Yet, a large proportion (~50%) of individuals who will go on to develop lung cancer are not currently screening-eligible. Notably, the current USPSTF criteria received a “B” recommendation, emphasizing the need for research to enhance the uptake of LDCT screening and develop biomarkers for more accurately identifying individuals at elevated risk of lung cancer who would benefit from screening [6]. Additionally, for individuals eligible for lung cancer screening (LCS), concerns persist regarding false-positive results and unnecessary follow-up procedures [3,5,6,7]. To this end, a recent study found that LCS in clinical practice had a major complication rate of 20.6%, which was significantly higher than the 9.4% major complication rate observed in the NLST [7].

Numerous lung cancer risk prediction models including Bach [8], Spitz [9], the Liverpool Lung Project (LLP) and Liverpool Lung Project Incidence (LLPi) Risk Models [10,11], Hoggart [12], PLCOm2012 [13], Pittsburgh [7], and the Lung Cancer Risk Assessment Tool (LCRAT) [14] were developed to identify individuals at high risk of lung cancer and who may benefit from LCS [7,8,10,11,12,13,14]. Additional models such as the Lung Cancer Death Risk Assessment Tool (LCDRAT) [14] and the Kovalchik model [15] predict lung cancer mortality. However, none of them have integrated any biomarkers into their frameworks.

Further improvements in lung cancer risk prediction may be achieved using biomarkers. Previously, our group established the merits of a four-marker protein panel (4MP) consisting of the precursor form of surfactant protein B (Pro-SFTPB) [8,9,10], cancer antigen 125 (CA125), carcinoembryonic antigen (CEA), and cytokeratin-19 fragment (CYFRA21-1). The Pro-SFTPB was found to result from the activation of the transcription factor NKX2.1, a known oncogene activated early during lung tumor development [10]. The four-marker protein panel is used for determining an individual’s risk of lung cancer among individuals who meet the current USPSTF screening criteria or with a history of smoking [10] or more pack years [16,17]. More recently, using pre-diagnostic sera from the Prostate, Lung, Colorectal, and Ovarian (PLCO) Cancer Screening Trial, we demonstrated that the 4MP together with the PLCOm2012 lung cancer risk model based on subject characteristics better identified individuals at high risk of a lethal lung cancer compared to the current USPSTF criteria [18].

In this study, using blinded pre-diagnostic plasmas collected from within 2 years of a lung cancer diagnosis from 25 cases and 100 age-, sex-, and smoking-matched controls from participants in the Physicians’ Health Study (PHS) cohort, we assessed the predictive performance of the 4MP for predicting lung cancer. The contributions of three additional LC-associated protein biomarkers, cancer antigen 15-3 (CA 15-3) [19,20], osteopontin (OPN) [20,21], and human epididymis protein 4 (HE4) [16], for improving upon the performance of the 4MP was also assessed. The performance of 4MP amongst never- and ever-smoking individuals was evaluated.

## 2. Materials and Methods

### 2.1. Physicians’ Health Study (PHS)

The Physicians’ Health Study (PHS) cohort comprises two groups: PHS I and II [11]. PHS I was initiated in 1982 and was a randomized, double-blind, placebo-controlled trial of 22,071 US male physicians aged 40–84 years aimed at evaluating the impact of aspirin and beta-carotene on cardiovascular disease (CVD) and cancer outcomes, respectively [22]. PHS II was a randomized, double-blind, placebo-controlled trial that followed in 1996 and that included 7641 PHS I participants plus an additional 7000 US male physicians aged ≥50 years to determine the impact of beta-carotene, vitamin C, vitamin E, and a daily multivitamin on the prevention of CVD, cancer, and other aging-related outcomes [23,24,25]. For both PHSI and II, all potentially eligible US male physicians identified from a roster provided by the American Medical Association were randomized into the intervention arm or placebo-control arm [11].

Written informed consent was obtained from each participant and the study was approved by the Human Research Committee at Brigham and Women’s Hospital. At baseline and prior to randomization, the PHS participants had their blood drawn, which was then fractionated by centrifugation and packed on dry ice for return within 24 h by overnight courier. Pre-randomization blood specimens were obtained from 14,916 (67.6%) of 22,071 PHS I participants and 11,133 (76.0%) of 14,641 PHS II participants. Upon receipt in the central laboratory, the blood components were immediately aliquoted, labeled, frozen, and stored at −82 °C for the PHS I samples and in liquid nitrogen at −170 °C for the PHS II samples. 

The morbidity and mortality outcomes for the PHS participants were determined through annual questionnaires and endpoint follow-up. The medical records were requested and obtained for newly reported self-reported cases of lung cancer for endpoint adjudication. The cases eligible for the current study were participants who (1) were cancer free at the baseline trial entry and who went on to develop medical record-confirmed lung cancer during follow-up and (2) had available baseline plasma samples for laboratory analyses. For each case, four controls who remained free of cancer during the study follow-up were randomly selected and matched to cases based on the date of recruitment into the cohort (±24 months), age at recruitment (±36 months), PHS I or II group, smoking status (never, former, or current), and among those currently smoking, categories of cigarettes smoked per day (1–19, 20–39, or 40 or more). For this study, the plasma samples consisted of 25 cases diagnosed with lung cancer and 100 participants without lung cancer, matched in terms of sex, age, and smoking history, as detailed earlier. The plasma samples were obtained from the cases collected within 2 years prior to the lung cancer diagnosis.

### 2.2. Assay of Protein Biomarkers

The plasma protein concentrations of CA15-3, CEA, CA125, CYFRA21-1, OPN, HE4, and Pro-SFTPB were determined via Luminex bead-based immunoassays using the Milliplex (MilliporeSigma, Burlington, MA, USA) kit HCCPBP1-58K according to the provided kit protocol. The concentrations for the Pro-SFTPB were determined using bead-based immunoassays on the MAGPIX^®^ instrument (Diasorin, Saluggia, Itay) as previously described [16,26,27]. The biomarker scores for the 4MP were calculated based on the previously developed logistic regression model [16,17]. The coefficients of variation amongst the pooled cases in the PHS cohort for CA15-3, CEA, CA125, CYFRA21-1, OPN, HE4, and Pro-SFTPB were 6.4%, 5.2%, 3.0%, 45.6%, 1.6%, 51.9%, and 3.8%, respectively. The coefficients of variation amongst the pooled healthy controls in the PHS cohort for CA15-3, CEA, CA125, CYFRA21-1, OPN, HE4, and Pro-SFTPB were 4.5%, 3.4%, 2.7%, 55.0%, 2.5%, 8.8%, and 17.5%, respectively.

### 2.3. Statistical Analyses

For these analyses, the 4MP scores were calculated based on a previously developed logistic regression model [16]. A logistic regression model was also used to assess the contributions of additional protein biomarkers (CA15-3, HE4, and OPN) on improving upon the performance of the 4MP. Here, we used two approaches. In the first approach, we developed a logistic regression model that considered the 4MP as a singular continuous variable with the composite 7MP model being as follows: −1.96 (intercept) + 1.28 (4MP score) − 0.41 (logCA15-3) − 0.14 (logHE4) − 0.53 (logOPN). In the second approach, we developed a logistic regression model that considered each of the 7 biomarkers as independent variables, with the composite model being as follows: −1.52 (intercept) + 0.79 (logProSFTPB) + 1.45 (logCEA) + 0.87 (logCA125) + 0.32 (logCYFRA21.1) − 0.22 (logCA15-3) − 0.21 (logHE4) − 0.68 (logOPN). We report the classifier performance for both approaches. 

The Area under the Receiver Operating Characteristic curves (AUC), sensitivity, and specificity estimates were determined for the individual protein biomarkers as well as for the 4MP and derived 7MP using the “pROC” package in R Statistical Software (https://www.r-project.org/, version 4.2.0, accessed on 22 May 2022). The evaluation of the AUC, sensitivity, and specificity was stratified into three time intervals: within 1 year, between 1 year and 2 years, and within two years prior to lung cancer diagnosis. Additionally, the AUC, sensitivity, and specificity for the biomarkers were further stratified into ever-smokers and never-smokers, as well as cases with metastatic lung cancer and those without metastatic lung cancer. The 95% confidence intervals (CIs) presented for the individual performance of each biomarker were based on the bootstrap procedure in which we resampled with replacement 1000 times for the cases and the corresponding matched controls. Apart from age and smoking status, no other confounding factors were considered.

The covariate-adjusted ROC curve (AROC) after adjustment for matching parameters (age and smoking status) was evaluated and the area under the AROC with 95% CI was estimated. The covariate-adjusted ROC analyses were performed with pcvsuite in R software (version 1.0) [28]. 

## 3. Results

### 3.1. Performance Estimates of the Four-Marker Protein Panel and Additional Protein Biomarkers for Risk Assessment of Lung Cancer in the PHS Cohort

The PHS included plasma collected within 2 years of an LC diagnosis from 25 cases as well as 100 smoking- and aged-matched non-case controls. The patient and tumor characteristics are provided in Table 1. Among the cases, 3 of them were non-smokers, 14 cases were past smokers, and 8 were current smokers. The mean age for the cases and matched controls was 69.4 and 69.2 years, respectively. The number of cases < 1 year and 1–2 years prior to a lung cancer diagnosis were 13 and 12, respectively.

The individual performance estimates (AUC) of the seven cancer-associated protein biomarkers for the 2-year risk of lung cancer ranged from 0.50 to 0.60 (Table S1). The performance estimates increased as the blood samples were taken closer to the diagnosis of LC (Table S1). 

**Figure 1:**
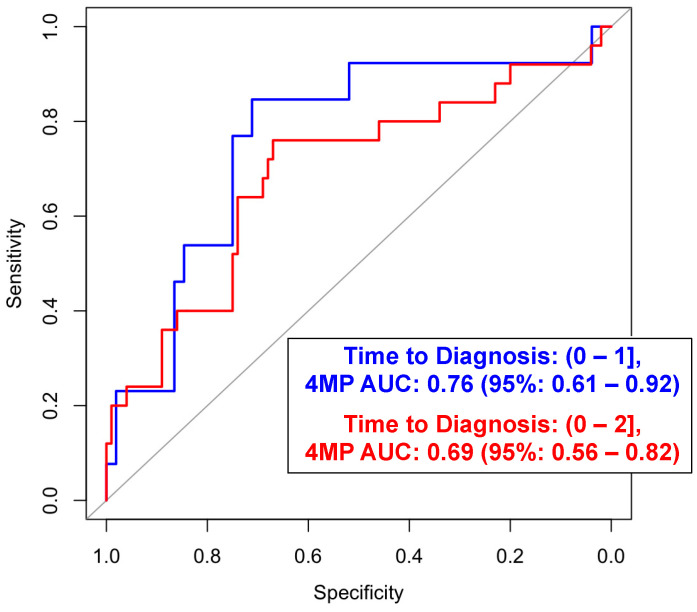
Predictive performance of the 4MP for predicting 1-year and 2-year lung cancer.

Among all the lung cancer cases, the performance estimates of the 4MP using fixed coefficients from the previously developed logistic regression model [16,27] yielded AUCs of 0.76 (95% CI: 0.61–0.92) and 0.69 (95% CI: 0.56–0.82) when considering the case plasma samples collected within 0–1 and 0–2 years prior to the diagnosis, respectively (Figure 1; Table S1). The addition of CA15-3, OPN, or HE4 did not yield statistically significant improvements for the risk prediction of LC compared to the 4MP alone. The corresponding AUC values within the periods of one year and two years prior to diagnosis were 0.78 (95% CI: 0.62–0.93) and 0.69 (95% CI: 0.57–0.82), respectively (Table S1). With the categorization of cases and controls into ever-smokers and never-smokers, the 4MP had respective AUCs of 0.77 (95% CI: 0.63–0.92) and 0.72 (95% CI: 0.17–1.00) for the 1-year risk prediction of lung cancer (Table 2 and Table S2), respectively. The AUC of the 4MP for ever-smokers within 2 years prior to a lung cancer diagnosis was 0.68 (95% CI: 0.54–0.82) (Table 2). The 4MP had an AUC of 0.81(95% CI: 0.57–1.00) in predicting 1–2-year lung cancer risk among current smokers (Table S2). An adjustment of performance based on the matching criteria showed a similar performance (Table S3). The absence of individuals who had never smoked within the case group and were diagnosed within 1 to 2 years of diagnosis precluded the evaluation of their performance. 

### 3.2. Performance Estimates of the Four-Marker Protein Panel for Metastatic Lung Cancer

**Figure 2:**
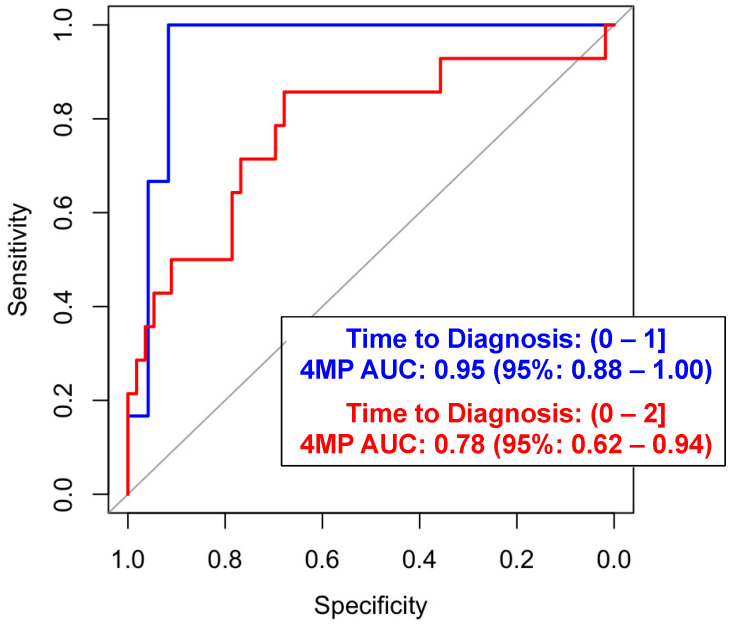
Predictive performance of the 4MP for predicting 1 year and 2 year of metastatic lung cancer.

Of the 25 LC cases, 14 (56%) presented with metastatic disease at the time of clinical diagnosis (Table 1). The 4MP yielded an AUC of 0.78 (95% CI: 0.62–0.94) with a sensitivity of 37.5% (95% CI: 0.07–0.64) given a specificity of 95% for predicting metastatic LC within 2 years of the blood draw and an AUC of 0.95 (95% CI: 0.88–1.00) with a sensitivity of 66.7% (0.95% CI: 0.10–1.00) at a specificity of 95% when considering the cases diagnosed within 1 year of the blood draw (Figure 2 and Figure S1, and Table S4).

## 4. Discussion

Numerous large-scale clinical trials have demonstrated the reduction in lung cancer mortality by low-dose CT-based screening. In the United States, the USPSTF currently recommends screening for individuals with ≥20 PYs smoking history, age ≥ 50, and quit date < 15 years ago. Participation in lung cancer screening in the United States has been low [12,13]. CT-based screening is gaining further acceptance in the world, but patient and provider concerns regarding the downstream procedures and complications associated with lung cancer screening are still a challenge [7,14]. A previous article substantiated that an integrated lung cancer risk model incorporating biomarkers and smoking exposure yielded an AUC of 0.83 in the European Prospective Investigation into Cancer and Nutrition (EPIC) and the Northern Sweden Health and Disease Study (NSHDS) [16]. Previously, we demonstrated the utility of the 4MP for individualized risk assessment of lung cancer with an AUC performance of 0.79 (95% CI: 0.77–0.82) for the case sera collected within one year preceding diagnosis and randomly selected non-case control in the Prostate, Lung, Colorectal and Ovarian (PLCO) screening trial [17]. A predictive model for lung cancer mortality risk, employing a combination of the 4MP and the PLCO_M2012_ score, demonstrated a one-year diagnostic AUC of 0.88 (95% CI: 0.86–0.90) with respective lung cancer death- and non-lung cancer death-specific hazard ratios of 10.65 and 3.27 [18]. In the current study, we assessed the extent by which the 4MP informs about the risk of lung cancer in a unique population of male physicians in the PHS cohort. We demonstrated that the 4MP is capable of identifying participants who would go on to be diagnosed with lung cancer, regardless of smoking status. 

Tobacco use is the leading cause of lung cancer, contributing to 55% of lung cancer deaths among women and greater than 70% of lung cancer deaths among men [15]. Moreover, mechanistically, tobacco contains several carcinogenic compounds that contribute to lung inflammation and promote tumorigenesis [16]. Several lines of evidence unequivocally support that smoking cessation reduces the risk of lung cancer and improves overall survival [17,18]. Moreover, smoking cessation decreases the quantity and dimensions of lung nodules, which are prevalent among individuals who have a history of smoking, while also enhancing lung function [19,20]. Thus, identifying individuals who have ever smoked and who are at elevated risk of lung cancer on the basis of the 4MP may serve as an impetus for enrollment into lung cancer screening programs for earlier detection as well as entry into smoking cessation programs. Yet, a larger proportion of lung cancer cases are diagnosed in never-smoker individuals with the increasing frequency of lung cancers in the past decades [29]. Specifically, it is estimated that lung cancer in those who have never smoked would be the fifth-leading cause of cancer mortality worldwide and the seventh-leading cause in the United States and accounts for up to 20% of lung cancer cases [21,22]. Lung cancer in those who have never smoked appears distinct from tobacco-related lung cancer in that it is more common in those of Asian descent and in women. Never-smoking lung cancer can show different premalignant progression, distribution of histological subtypes, driver mutation frequencies, stage at diagnosis, and prognosis [21]. While risk factors like radon, asbestos, and second-hand smoke exposure and family history have been evaluated as risk factors, these factors are only minor contributors in relation to active tobacco smoking [23]. In Asian populations where lung cancer amongst never-smokers is a greater public health concern, screening is being broadened to those with lower risk [24]. In Taiwan, national screening guidelines include those with no smoking history who have additional risk factors for lung cancer, including family history [25]. However, there is a great concern that broadening screening in a lower-incidence population will increase false-positive tests, the overdiagnosis of lung cancer, and health care utilization without improving overall mortality [26,27]. Indeed, early analyses of national screening programs in Asia that have included lower risk individuals have demonstrated dramatic increases in early stage lung cancer diagnoses and reduced late-stage lung cancer incidence without the change in mortality outcome, suggesting high rates of overdiagnosis [27,28].

The present study showed that our proposed combination of four biomarkers has stable performance amongst never-smoker individuals, suggesting an opportunity to consider an expansion of screening-eligibility criteria beyond age and smoking status to better capture at-risk individuals.

The 4MP appeared to be most predictive amongst participants who developed metastatic lung cancer. Detecting earlier metastatic lung cancer may provide a stage-shifting benefit with prolonged survival outcomes [29]. Therefore, our findings reinforce the potential clinical benefit of blood-based biomarkers for the risk assessment of lung cancer to inform about the need for screening. 

### Limitations

The limitations in this study include the small sample size and limited information regarding mortality outcomes. Nevertheless, the performance of the 4MP is consistent with our previously reported validation efforts [16,17,26] and the 4MP informs on the risk of lethal lung cancers in the pre-diagnostic PLCO cohort [29]. Additionally, few women had the opportunity to graduate from medical school, precluding the inclusion of women in the study in 1982. As a result, the 4MP was only evaluated in white male physicians in this study and the performance of the 4MP amongst women and other ethnicities remains to be determined. Furthermore, the high coefficient of variations for CYFRA21-1 and HE4 underscores the need for further validation of the respective assays. 

## 5. Conclusions

The 4MP has the potential to identify individuals at high risk of a subsequent diagnosis of lung cancer, even when the disease may begin to develop many years before clinical diagnosis. This early identification can provide a valuable two-year head start in the treatment of lung cancer, allowing for timely interventions that significantly enhance the likelihood of successful outcomes. By better selecting individuals who would benefit from screening, the 4MP not only addresses the extended latent period of lung cancer but also underscores the critical value of early intervention and improved patient outcomes.

## Figures and Tables

**Table 1:** Baseline characteristics of Physicians’ Health Study participants.

		Controls (*n* = 100)	Lung Cancer Cases (*n* = 25)
		N (%)	N (%)
Smoking Status	Never	12 (12)	3 (12)
	Past	56 (56)	14 (56)
	Current	32 (32)	8 (32)
Metastatic Status	Non-Metastatic		11 (44)
	Metastatic		14 (56)
Time to Diagnosis	0–1 yr		13 (52)
	1–2 yr		12 (48)
Histology	Not available		2 (8)
	Neoplasm, Malignant		1 (4)
	Carcinoma, NOS		4 (16)
	Small Cell Carcinoma		2 (8)
	Squamous Cell Carcinoma		6 (24)
	Adenocarcinoma		9 (36)
	Alveolar Adenocarcinoma		1 (4)
		Mean, Median (IQR)	
Age at blood draw date		69.16, 70.08 (65.95, 74.58)	69.43, 70.10 (66.28, 75.65)
Pro-SFTPB (ng/mL)		51.27, 35.39 (14.43, 61.63)	98.06, 47.25 (25.44, 81.77)
CEA (ng/mL)		1.09, 0.93 (0.56, 1.44)	4.44, 1.37 (0.58, 3.41)
CA125 (U/mL)		3.36, 2.77 (2.24, 4.04)	5.27, 3.32 (2.36, 4.50)
CYFRA-21-1 (ng/mL)		0.20, 0.05 (0.02, 0.21)	0.35, 0.09 (0.02, 0.60)
CA15-3 (U/mL)		17.65, 15.12 (9.31, 20.47)	23.08, 13.94 (11.06, 24.07)
OPN (ng/mL)		26.68, 24.34 (15.73, 31.96)	26.48, 26.18 (17.48, 33.22)
HE4 (ng/mL)		10.17, 2.53 (0.75, 7.65)	5.15, 4.69 (2.71, 8.41)

**Table 2:** Performance evaluation of the four-marker protein panel stratified into never- and ever-smokers.

	AUC Performance of 4MP (95% CI)	
Time to DX	Never-Smokers	Ever-Smokers
[0–1)	0.72 (0.17–1.00)	0.77 (0.63–0.92)
[1–2)	-	0.59 (0.38–0.80)
[0–2)	0.72 (0.17–1.00)	0.68 (0.54–0.82)

## Data Availability

The supporting data for the findings of this study can be found in the Article and Supplemental Materials. Additional information is available from the authors upon request.

## Supplementary Materials

The following supporting information can be downloaded at: https://www.mdpi.com/article/10.3390/cancers16112070/s1, Figure S1. Predictive performance of the 4MP for predicting 1-year and 2-year non-metastatic lung cancer; Table S1. Performance evaluation of the protein markers in the Physicians’ Health Study (PHS); Table S2. Performance evaluation of the four-marker protein panel stratified into never-, past- and current-smokers; Table S3. Adjusted performance evaluation of the markers in the PHS cohort; Table S4. Performance evaluation of the markers in the PHS cohort stratified into metastatic and non-metastatic lung cancer.
