# Supplementary material for: Validation of a Blood-Based Protein Biomarker Panel for a Risk Assessment of Lethal Lung Cancer in the Physicians’ Health Study
Source: Cancers (Basel). 2024 May 30;16(11):2070. doi: 10.3390/cancers16112070 (PMC11171146; doi:10.3390/cancers16112070)
Supplement: Supplementary file 1 [file cancers-16-02070-s001.zip › cancers-2800101-supplementary.pdf]

Appendix A

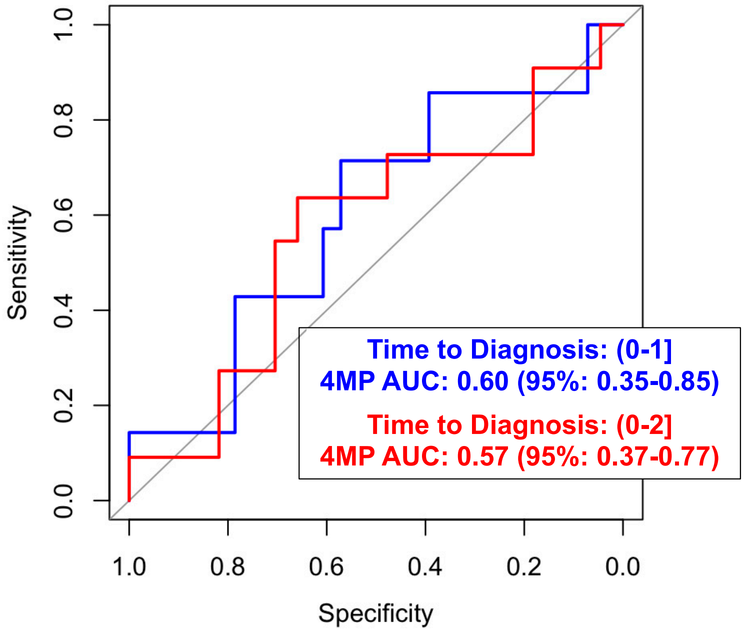

Figure S1. Predictive performance of the 4MP for predicting 1-year and 2-year non-metastatic lung cancer.

Table S1. Performance evaluation of the protein markers in the Physicians' Health Study (PHS)

		AUC performance (95% CI)									
**Time to DX**	**Numbers**	**CA125**	**CEA**	**CYFRA21.1**	**ProSFTPB**	**4MP[1]**	**CA153**	**OPN**	**HE4**	**7MP[1] (4MP + CA125 + OPN + HE4)**	**7MP (CA125 + CEA + CYFRA21 + ProSFTPB + CA125 + OPN + HE4)**
[0-1)	Ncases: 13 Ncontrols: 52	0.70 (0.53-0.87)	0.60 (0.37-0.83)	0.72 (0.57-0.87)	0.57 (0.39-0.75)	0.76 (0.61-0.92)	0.51 (0.34-0.69)	0.62 (0.46-0.78)	0.63 (0.49-0.77)	0.78 (0.62-0.93)	0.74 (0.56-0.92)
[1-2)	Ncases: 12 Ncontrols: 48	0.44 (0.24-0.64)	0.55 (0.33-0.77)	0.44 (0.22-0.65)	0.63 (0.43-0.83)	0.59 (0.38-0.80)	0.55 (0.35-0.75)	0.38 (0.19-0.58)	0.50 (0.32-0.68)	0.58 (0.37-0.79)	0.58 (0.36-0.79)
[0-2)	Ncases: 25 Ncontrols: 100	0.56 (0.43-0.70)	0.59 (0.44-0.74)	0.59 (0.46-0.72)	0.60 (0.47-0.73)	0.69 (0.56-0.82)	0.53 (0.40-0.67)	0.50 (0.38-0.63)	0.57 (0.45-0.69)	0.69 (0.57-0.82)	0.68 (0.54-0.81)

[1] 4MP were treated as a fixed score derived previously.

Table S2. Performance evaluation of the 4-marker protein panel stratified into never-, past- and current-smokers.

Time to diagnosis		
**[0-1)**	**[1-2)**	**[0-2)**

	Numbers	AUC performance of 4MP (95% CI)	Numbers	AUC performance of 4MP (95% CI)	Numbers	AUC performance of 4MP (95% CI)
Never-smokers	Ncases: 3<br>Ncontrols: 12	0.72<br>(0.17-1.00)	Ncases: 0<br>Ncontrols: 0	-	Ncases: 3<br>Ncontrols: 12	0.72<br>(0.17 - 1.00)
Past-smokers	Ncases: 6<br>Ncontrols: 24	0.88<br>(0.75 - 1.00)	Ncases: 8<br>Ncontrols: 32	0.48<br>(0.21-0.74)	Ncases: 14<br>Ncontrols: 56	0.67<br>(0.48 - 0.85)
Current-smokers	Ncases: 4<br>Ncontrols: 16	0.65<br>(0.16-0.96)	Ncases: 4<br>Ncontrols: 16	0.81<br>(0.57-1.00)	Ncases: 8<br>Ncontrols: 32	0.69<br>(0.46 - 0.92)

Table S3. Adjusted performance evaluation of the markers in PHS cohort.

		AUC performance (95% CI)									
Time to DX	Numbers	CA125	CEA	CYFRA21.1	ProSFTPB	4MP[1]	CA153	OPN	HE4	7MP[1] (4MP + CA125 + OPN + HE4)	7MP (CA125 + CEA + CYFRA21 + ProSFTPB + CA125 + OPN + HE4)
[0-1)	Ncases: 13<br>Ncontrols: 52	0.68<br>(0.50-0.84)	0.66<br>(0.44-0.86)	0.67<br>(0.46- 0.84)	0.52<br>(0.34-0.71)	0.75<br>(0.57-0.91)	0.48<br>(0.28-0.68)	0.56<br>(0.36-0.75)	0.56<br>(0.36-0.75)	0.75<br>(0.56-0.91)	0.76<br>(0.54- 0.93)
[1-2)	Ncases: 12<br>Ncontrols: 48	0.42<br>(0.24-0.64)	0.58<br>(0.38-0.77)	0.47<br>(0.26- 0.68)	0.63<br>(0.42-0.81)	0.55<br>(0.35-0.76)	0.54<br>(0.35-0.73)	0.40<br>(0.22-0.58)	0.46<br>(0.27-0.67)	0.58<br>(0.37-0.79)	0.58<br>(0.38- 0.79)
[0-2)	Ncases: 25<br>Ncontrols: 100	0.56<br>(0.40-0.68)	0.63<br>(0.49-0.77)	0.58<br>(0.45- 0.70)	0.58<br>(0.44-0.71)	0.66<br>(0.53-0.80)	0.53<br>(0.40-0.66)	0.49<br>(0.35-0.62)	0.56<br>(0.41-0.66)	0.68<br>(0.54-0.81)	0.69<br>(0.55- 0.83)

[1] 4MP were treated as a fixed score derived previously.

Table S4. Performance evaluation of the markers in PHS cohort stratified into metastatic and non-metastatic lung cancer.

		AUC performance (95% CI)									
Time to DX	Numbers	CA125	CEA	CYFRA21.1	ProSFTPB	4MP	CA153	OPN	HE4	7MP[1] (4MP + CA125 + OPN + HE4)	7MP (CA125 + CEA + CYFRA21 + ProSFTPB + CA125 + OPN + HE4)
						Non-Metastatic lung cancer					
[0-1)	Ncases: 7<br>Ncontrols: 28	0.61<br>(0.34-0.87)	0.57<br>(0.22-0.91)	0.58<br>(0.34-0.82)	0.48<br>(0.23-0.73)	0.60<br>(0.35-0.85)	0.46<br>(0.24-0.69)	0.52<br>(0.31-0.72)	0.58<br>(0.38-0.77)	0.61<br>(0.36-0.86)	0.61<br>(0.32-0.90)
[1-2)	Ncases: 4<br>Ncontrols: 16	0.41<br>(0.1-0.71)	0.50<br>(0.09-0.91)	0.32<br>(0.07-0.57)	0.58<br>(0.24-0.92)	0.56<br>(0.18-0.95)	0.53<br>(0.16-0.9)	0.50<br>(0.19-0.81)	0.34<br>(0.07-0.62)	0.53<br>(0.2-0.86)	0.53<br>(0.24-0.83)
[0-2)	Ncases: 11<br>Ncontrols: 44	0.55<br>(0.34-0.75)	0.56<br>(0.32-0.8)	0.49<br>(0.3-0.69)	0.49<br>(0.3-0.69)	0.57<br>(0.37-0.77)	0.48<br>(0.3-0.67)	0.51<br>(0.35-0.68)	0.52<br>(0.35-0.7)	0.57<br>(0.38-0.77)	0.57<br>(0.36-0.78)
						Metastatic lung cancer					
[0-1)	Ncases: 6<br>Ncontrols: 24	0.81<br>(0.63-0.99)	0.66<br>(0.33-0.98)	0.88<br>(0.73-1.00)	0.70<br>(0.48-0.92)	0.95<br>(0.87-1.00)	0.57<br>(0.28-0.86)	0.73<br>(0.45-1.00)	0.69<br>(0.48-0.91)	0.97<br>(0.90-1.00)	0.92<br>(0.79-1.00)
[1-2)	Ncases: 8<br>Ncontrols: 32	0.45<br>(0.18-0.71)	0.59<br>(0.32-0.86)	0.47<br>(0.18-0.75)	0.68<br>(0.43-0.94)	0.62<br>(0.35-0.89)	0.56<br>(0.32-0.81)	0.35<br>(0.09-0.60)	0.54<br>(0.31-0.77)	0.65<br>(0.37-0.94)	0.63<br>(0.34-0.92)

[0-2)	Ncases: 14<br>Ncontrols: 56	0.57<br>(0.38-0.76)	0.62<br>(0.43-0.82)	0.65<br>(0.47-0.84)	0.67<br>(0.50-0.84)	0.78<br>(0.62-0.94)	0.56<br>(0.38-0.75)	0.50<br>(0.31-0.69)	0.60<br>(0.44-0.76)	0.80<br>(0.64-0.97)	0.77<br>(0.60-0.94)

[1] 4MP were treated as a fixed score derived previously.
